# PET Imaging for Oxidative Stress in Neurodegenerative Disorders Associated with Mitochondrial Dysfunction

**DOI:** 10.3390/antiox9090861

**Published:** 2020-09-14

**Authors:** Masamichi Ikawa, Hidehiko Okazawa, Yasunari Nakamoto, Makoto Yoneda

**Affiliations:** 1 Second Department of Internal Medicine, Faculty of Medical Sciences, University of Fukui, Fukui 910-1193, Japan; iqw@u-fukui.ac.jp (M.I.); ynakamot@u-fukui.ac.jp (Y.N.); 2 Biomedical Imaging Research Center, University of Fukui, Fukui 910-1193, Japan; 3 Department of Advanced Medicine for Community Healthcare, Faculty of Medical Sciences, University of Fukui, Fukui 910-1193, Japan; 4 Faculty of Nursing and Social Welfare Science, Fukui Prefectural University, Fukui 910-1195, Japan; myoneda@fpu.ac.jp

**Keywords:** amyotrophic lateral sclerosis, mitochondrial disease, mitochondrial dysfunction, neurodegenerative disorder, oxidative stress, Parkinson’s disease, positron emission tomography

## Abstract

Oxidative stress based on mitochondrial dysfunction is assumed to be the principal molecular mechanism for the pathogenesis of many neurodegenerative disorders. However, the effects of oxidative stress on the neurodegeneration process in living patients remain to be elucidated. Molecular imaging with positron emission tomography (PET) can directly evaluate subtle biological changes, including the redox status. The present review focuses on recent advances in PET imaging for oxidative stress, in particular the use of the Cu-ATSM radioligand, in neurodegenerative disorders associated with mitochondrial dysfunction. Since reactive oxygen species are mostly generated by leakage of excess electrons from an over-reductive state due to mitochondrial respiratory chain impairment, PET with ^62^Cu-ATSM, the accumulation of which depends on an over-reductive state, is able to image oxidative stress. ^62^Cu-ATSM PET studies demonstrated enhanced oxidative stress in the disease-related brain regions of patients with mitochondrial disease, Parkinson’s disease, and amyotrophic lateral sclerosis. Furthermore, the magnitude of oxidative stress increased with disease severity, indicating that oxidative stress based on mitochondrial dysfunction contributes to promoting neurodegeneration in these diseases. Oxidative stress imaging has improved our insights into the pathological mechanisms of neurodegenerative disorders, and is a promising tool for monitoring further antioxidant therapies.

## 1. Introduction

In neurodegenerative disorders such as Parkinson’s disease, amyotrophic lateral sclerosis (ALS), and Alzheimer’s disease, increasing evidence from pathological and biochemical studies suggests that oxidative stress and mitochondrial dysfunction are the principal molecular mechanisms in the neurodegenerative process [1,2]. Since impaired mitochondria are a major source of reactive oxygen species (ROS) [3,4], oxidative stress is closely linked to mitochondrial dysfunction and has been assumed to play a crucial role in the pathogenesis of neurodegenerative disorders associated with mitochondrial dysfunction [5]. Indeed, many studies demonstrated an increase in oxidized molecules, reduced antioxidant capacity, and impaired mitochondrial metabolism in various neurodegenerative disorders [2,6,7]. In addition, both oxidative stress and mitochondrial dysfunction induce aggregation of misfolded proteins, such as amyloid-β, tau, and α-synuclein, which is the primary pathogenesis of neurodegenerative disorders [8,9].

While direct and non-invasive evaluation of the cerebral redox status is difficult to perform in living patients, molecular imaging technology with positron emission tomography (PET) has enabled visualization of oxidative stress in the brain [10,11,12,13]. PET imaging allows us to evaluate minuscule changes in biological phenomena and processes, such as receptor distribution and metabolic activity, at the molecular level by the administration of specific radioligands [14]. Our recent studies with PET imaging demonstrated increased oxidative stress in the disease-related brain regions of patients with neurodegenerative disorders associated with mitochondrial dysfunction, including mitochondrial disease, Parkinson’s disease, and ALS [10,11,12,13].

In this review, we focus on the recent achievements and future potential prospects of PET imaging for oxidative stress in neurodegenerative disorders.

## 2. Oxidative Stress and Mitochondrial Dysfunction

### 2.1. Oxidative Stress Caused by Reactive Oxygen Species

Oxidative stress is classically defined as an imbalanced redox state in which the oxidation effect caused by increased ROS production exceeds the defense capacity of the antioxidant mechanism [5]. Enhanced oxidative stress due to excess ROS generation leads to oxidative damage to the cellular components, such as proteins, lipids, and DNA. Additionally, ROS change the expression of nuclear factor kappa B (NF-κB), a transcription factor responsible for inducing inflammation and apoptosis [15]. These ROS-induced pathological mechanisms provoke tissue and organ dysfunction, especially neuronal degeneration in the brain [6,7]. ROS such as superoxide (O_2_^−^), hydroxyl radical (OH), and hydrogen peroxide (H_2_O_2_), are derived from molecular oxygen by the reduction. In particular, superoxide and hydroxyl radical are classified as free radicals, which show high chemical reactivity due to their unpaired electrons [3]. ROS are endogenously produced in the mitochondria, peroxisomes, and endoplasmic reticulum of cells [16]. Among these organelles, mitochondria, which consume more than 90% of intravital oxygen during oxidative phosphorylation (i.e., the aerobic metabolism), are regarded as the principal endogenous source of ROS [17,18]. However, under a healthy condition with normal mitochondrial function, the amount of ROS leakage is so small that it can be eliminated by the endogenous biological antioxidants, such as superoxide dismutase (SOD) and glutathione (GSH) [5,19].

### 2.2. Mitochondria as a Major Source of Reactive Oxygen Species

The mitochondrion is an organelle that produces adenosine triphosphate (ATP) as energy essential for life activities using the intrinsic respiratory chains. In the mitochondrial respiratory chains (a.k.a. electron transport chains), which consist of five complexes (i.e., complex I-V), electrons obtained as the reduced form of nicotinamide adenine dinucleotide (NADH) from the metabolism of glucose (i.e., glycolysis), free fatty acids (i.e., β-oxidation), and the tricarboxylic acid cycle are transported to synthesize ATP [3,20,21]. Most of the transferred electrons are ultimately captured by oxygen in the four-electron reduction whereby electrons and oxygen are detoxified to harmless and stable water molecules [22]. However, in respiratory chain impairment due to mitochondrial dysfunction, deteriorated electron transport provokes excessive accumulation of electrons relative to the amount of oxygen, resulting in an over-reductive state [23,24]. Because ROS are produced by the reduction of molecular oxygen, redundant electrons that leak from the impaired respiratory chains in an over-reductive state readily react with oxygen, which generates ROS [4,25]. A total of nine sites have been identified as the sources of mitochondrial ROS; complex I produces superoxide solely in the matrix, while complex III generates superoxide in both the matrix and the intermembrane space [26]. As explained above, mitochondrial respiratory chain impairment provokes an over-reductive state, and this state under the normoxic condition results in oxidative stress, which suggests that the evaluation of an over-reductive state using molecular imaging would be a promising marker for oxidative stress.

### 2.3. Oxidative Stress Based on Mitochondrial Dysfunction in Neurodegenerative Disorders

As mentioned above, mitochondrial respiratory chain impairment causes oxidative stress due to an over-reductive state, in addition to an ATP production deficit [4]. Since mitochondria are distributed throughout the body, mitochondrial dysfunction may cause failures of various organs. In particular, the brain consumes 20% of intravital oxygen and has a relatively fragile antioxidant capacity [27,28], which underlies the vulnerability of the neurons and glial cells to oxidative stress due to mitochondrial dysfunction [18,29]. Besides reduced respiratory capacity of mitochondria, there are other possible causes of oxidative stress in the brain, e.g., neuroinflammation, protein aggregation, and decreased antioxidant defenses [7,30]. Aging is also a major factor in promoting these pathological mechanisms, especially decreased mitochondrial function and antioxidant potential, leading to the enhancement of cerebral oxidative stress in elderly people [17]. These factors explain why the prevalence of neurodegenerative disorders increases with advancing age, and many pathological and biochemical studies have demonstrated enhanced oxidative stress in various neurodegenerative disorders [1,2]. Interestingly, basic studies showed that aggregated misfolded proteins induce mitochondrial dysfunction and ROS generation [31,32]. Conversely, ROS may facilitate neurotoxic protein aggregation, such as amyloid-β (in AD), α-synuclein (in PD) and SOD1 (in ALS), as well as mitochondrial impairment, producing a vicious cycle among oxidative stress, mitochondrial dysfunction and protein aggregation [8,9]. These findings may indicate that the increase in ROS production precedes the appearance of plaque deposits and that mitochondrial dysfunction can be an early event that precedes protein aggregation in neurodegenerative disorders. [1,33,34].

## 3. Oxidative Stress Imaging with ^62^Cu-ATSM PET

### 3.1. Accumulation Mechanism of ^62^Cu-ATSM

**Figure 1:**
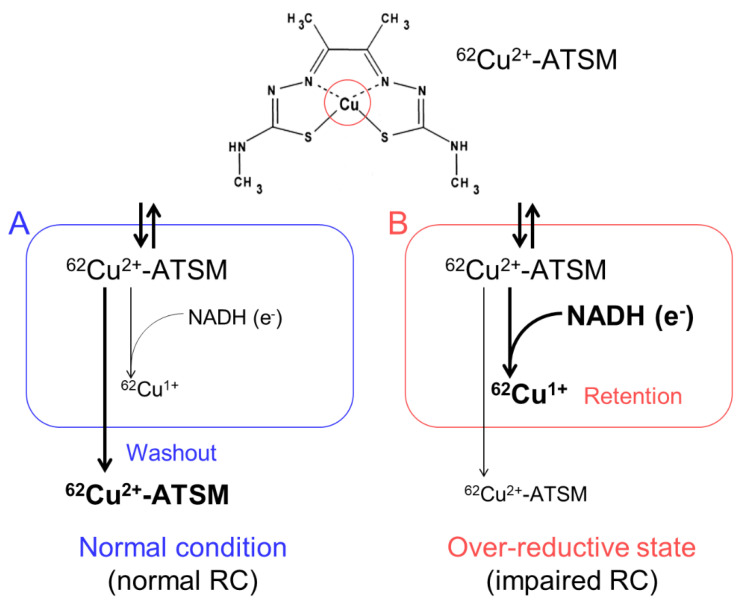
The accumulation mechanism of ^62^Cu-ATSM: ^62^Cu-ATSM contains a radioactive divalent copper (^62^Cu^2+^) in the complex (circle in the upper panel). This radioligand is distributed by the blood flow after intravenous administration and readily penetrates cells. The distributed ^62^Cu-ATSM is rapidly washed out from the cells under the normal condition with intact mitochondria (**A**). In contrast, in sites with excess electrons supplied as the reduced form of nicotinamide adenine dinucleotide (NADH) (i.e., an over-reductive state) due to mitochondrial dysfunction, ^62^Cu-ATSM is retained in cells by a reduction of Cu^2+^ to Cu^1+^ (**B**). RC, mitochondrial respiratory chain.

We developed an imaging technique for oxidative stress due to an over-reductive state using PET with ^62^Cu-diacetyl-bis(*N*^4^-methylthiosemicarbazone) (^62^Cu-ATSM), and succeeded in demonstrating increased oxidative stress in the brains of living patients with various neurodegenerative disorders [10,11,12,13]. ^62^Cu-ATSM, a PET radioligand, is a chelate complex that contains a radioactive divalent copper (^62^Cu^2+^) [35] (Figure 1). ^62^Cu-ATSM has high membrane permeability owing to its high lipophilicity and low molecular weight, allowing it to cross the blood-brain barrier [35]. The ^62^Cu^2+^ in this radioligand has two aspects. One is radioactivity (^62^Cu); the ^62^Cu emits positrons by β^+^ decay. The accumulation site of ^62^Cu-ATSM can be visualized by a PET scanner that captures the annihilation radiation pairs of the positrons. The other is divalence (Cu^2+^); due to reduction of the divalent form (Cu^2+^) to the monovalent form (Cu^1+^) in sites with excess electrons (i.e., an over-reductive state), the reduced Cu^1+^ dissociates from the ATSM complex and is irreversibly retained in the sites [35,36,37,38,39].

Several radioactive copper isotopes besides ^62^Cu, such as ^60^Cu, ^61^Cu, ^64^Cu, and ^67^Cu, can be used for labeling this ligand. These isotopes have different half-lives and decay modes, and thus the ligand can be adapted to the purpose and setting [40]. The distribution of Cu-ATSM is not affected by the type of the labeled copper isotopes, except for the background activity which depends on the time required for ligand washout [38,41,42].

Cu-ATSM was originally developed to visualize hypoxic regions [35]. Since an over-reductive state is one in which cells and tissues have excessive levels of electrons relative to oxygen, both hypoxia (i.e., relatively decreased oxygen) and impaired respiratory chains (i.e., relatively increased electrons) induce this state [35,43,44]. Indeed, many studies have shown the utility of Cu-ATSM to image tumor hypoxia or ischemic myocardium [36,37,38,39,43,44,45,46,47,48]. While Cu-ATSM can be available as a hypoxia imaging ligand, several basic studies indicated that accumulation of Cu-ATSM also occurs in normoxic tissue according to the presence of an intracellular over-reductive state with high NADH concentrations [24,35,49,50,51] (see also Section 4.2). Since neuronal degeneration is basically a non-hypoxic process, the cerebral accumulation of Cu-ATSM in patients with neurodegenerative disorders is assumed to depend on an over-reductive state caused mainly by reduced respiratory capacity of mitochondria, which results in oxidative stress [13].

### 3.2. PET Procedure with ^62^Cu-ATSM

^62^Cu-ATSM is obtained by simple mixing of generator eluate (^62^Cu-glycine) and ATSM solution (0.5 mM in dimethyl sulfoxide) [11,35,47,52]. The radiochemical purity of ^62^Cu-ATSM is confirmed by high-performance liquid chromatography (HPLC). ^62^Cu is eluted from a ^62^Zn/^62^Cu positron generator; the half-lives of ^62^Cu and the parent ^62^Zn are approximately 10 min and 9 h, respectively. Owing to the shorter half-life of ^62^Cu and the longer half-life of the parent ^62^Zn, ^62^Cu-ATSM can be eluted from a generator system every hour, permitting repeated PET scans during a whole day in clinical use [35,36].

PET scans with ^62^Cu-ATSM for the whole brain are performed for dynamic frames of 20 min or longer after intravenous bolus injection of 444-740 MBq ^62^Cu-ATSM [10,11,12,52]. The obtained PET data are usually reconstructed into static images in two phases: Early (up to 3 min) and delayed (10-20 min after injection). They are then converted into semiquantitative images with a unit of standardized uptake value (SUV), which is the tissue radioactivity concentration normalized by the injection dose and body weight (tissue concentration of radioactivity/[injection dose/body weight]) [13].

After intravenous injection, ^62^Cu-ATSM is distributed throughout the body including the brain by the blood flow in the early phase within 3 min. This radioligand readily penetrates cells but is rapidly washed out under normal conditions [10,35,36,52]. Contrastingly, in sites with an over-reductive state, this radioligand is retained in the delayed phase, approximately 7 min after injection [10,44]. Based on the aforementioned distribution property of ^62^Cu-ATSM, the accumulation of ^62^Cu-ATSM in early-phase images (obtained up to 3 min after injection) represents the blood flow distribution in the brain, whereas its accumulation in delayed-phase images (obtained 10–20 min after injection) reflects an over-reductive state [10,11,12,52]. Thus, in neurodegenerative disorders, delayed-phase images of ^62^Cu-ATSM PET indicate increased oxidative stress due to an over-reductive state caused mainly by mitochondrial dysfunction.

## 4. Oxidative Stress Imaging in Mitochondrial Disease

### 4.1. Oxidative Stress in Mitochondrial Diseases

Mitochondria incorporate mitochondrial DNA (mtDNA) as its own genome, which partially encodes the enzyme complexes of the respiratory chain [53]. Thus, mutations in mtDNA may induce mitochondrial dysfunction with impaired respiratory chains, which causes inherited mitochondrial diseases [54]. Among the mitochondrial diseases with mtDNA mutations, mitochondrial myopathy, encephalopathy, lactic acidosis, and stroke-like episodes (MELAS) syndrome (OMIM 540000) is one of the most common phenotypes and is mainly caused by the A-to-G transition mutation at nucleotide position 3243 in mtDNA (m.3243A>G) [55]. Various characteristic symptoms, such as stroke-like episodes (SEs), myopathy, cardiomyopathy, diabetes, and hearing loss, are frequently observed in patients with MELAS syndrome [56].

Since mitochondrial dysfunction results in increased ROS production, enhancement of oxidative stress is assumed in mitochondrial diseases including MELAS [57], and increased oxidative stress in the blood of patients with MELAS has been observed [58]. Recent in vitro studies also showed respiratory defects and increased ROS generation in human cell lines (cybrids) carrying mutated mitochondria derived from a patient with MELAS harboring m.3243A>G [4,59], which suggests that oxidative stress is closely associated with the pathogenesis of mitochondrial diseases.

### 4.2. In Vitro Studies with ^64^Cu-ATSM in MELAS Model Cells

To demonstrate the feasibility of ^62^Cu-ATSM as a PET radioligand for imaging oxidative stress due to an over-reductive state based on mitochondrial dysfunction, in vitro and in vivo studies were performed on MELAS cybrids [10,24]. Since cybrids harboring m.3243A>G closely replicate the pathophysiological conditions of MELAS [4,59], the in vitro accumulation of ^64^Cu-ATSM, having a longer half-life (13 h) than ^62^Cu, was evaluated in MELAS cybrids with an intracellular over-reductive state caused by mitochondrial respiratory defects [24,51]. These in vitro studies showed 1.5-fold increased retention of ^64^Cu-ATSM in the MELAS cybrids compared with wild-type cybrids having normal mitochondria under normoxia. ^64^Cu-ATSM retention significantly increased with the intracellular levels of NADH, the biological reductant as an electron donor, in the cybrids, which suggests that Cu-ATSM accumulation reflects an intracellular over-reductive state caused by mitochondrial dysfunction [24]. Since ROS generation is enhanced in the MELAS cybrids with an over-reductive state [4,59], increased Cu-ATSM accumulation indicates enhanced oxidative stress due to an over-reductive state based on mitochondrial dysfunction.

### 4.3. PET Imaging for Oxidative Stress in Patients with Stroke-Like Episodes of MELAS

Alongside the above in vitro studies, PET imaging with ^62^Cu-ATSM was performed in a patient with MELAS carrying m.3243A>G who had brain lesions caused by SEs, to evaluate the clinical utility of ^62^Cu-ATSM PET for detecting cerebral oxidative stress based on mitochondrial dysfunction in vivo [10].

SEs occur repeatedly and determine the prognosis of patients with MELAS [56,60]. Several pathophysiological hypotheses have been proposed for SEs [61]: Mitochondrial angiopathy (endothelial dysfunction in cerebral blood vessels) [62,63], cytopathy (neuronal dysfunction due to mitochondrial metabolic failure) [64], and neuronal hyperexcitability [65]. The pathological findings, such as an increased number of abnormal mitochondria in the endothelial cells and disruption of the endothelial tight junctions in SE lesions [62,66], support these hypotheses. In addition, recent studies using magnetic resonance (MR) imaging with ^1^H-MR spectroscopy, apparent diffusion coefficient (ADC) maps, MR angiography, and arterial spin labeling (ASL) methods showed hyperperfusion and vasogenic edema with vasodilatation and lactic acid fermentation in acute lesions of SEs [67,68,69,70,71], which also suggests the involvement of mitochondrial angiopathy and cytopathy in the pathogenesis of SEs. Moreover, PET imaging with ^15^O tracers demonstrated a significant reduction in both the oxygen extraction fraction (OEF) and cerebral metabolic rate of oxygen (CMRO_2_) in acute SE lesions [72], which also indicates impaired mitochondrial oxygen consumption (i.e., mitochondrial cytopathy). Besides these pathophysiological hypotheses, oxidative stress caused by mitochondrial dysfunction has been assumed to play a crucial role in the pathogenesis of SEs [57]. A postmortem study showing an increase in the level of 8-hydroxy-2′-deoxyguanosine (8-OHdG; DNA oxides) in SE lesions supports this assumption [73].

**Figure 2:**
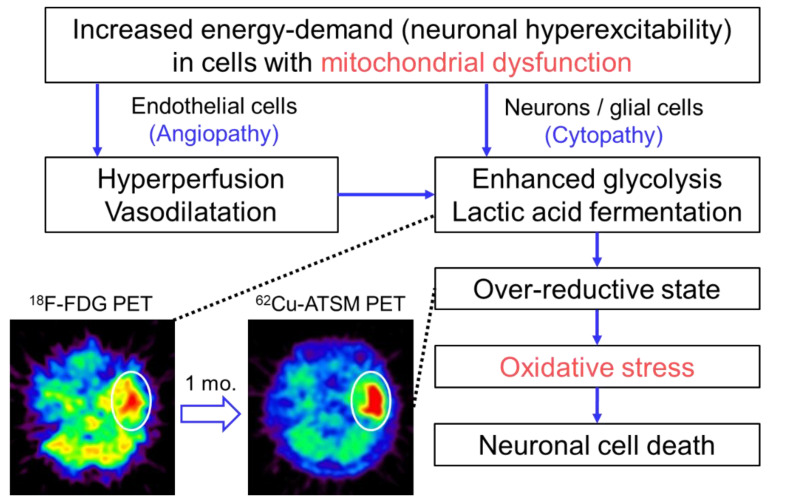
The pathophysiological process of stroke-like episodes (SEs) in mitochondrial encephalopathy, lactic acidosis, and stroke-like episodes (MELAS) hypothesized based on imaging studies. Brain positron emission tomography (PET) with ^18^F-FDG exhibited increased uptake in the acute SE lesion (circle) in a patient with MELAS who had an SE attack immediately before the PET scan. Interestingly, the lesion showed increased accumulation of ^62^Cu-ATSM on PET imaging (circle) performed one month after the SE attack (i.e., subacute phase). These imaging findings suggest that enhanced glycolysis is followed by increased oxidative stress due to an over-reductive state based on mitochondrial dysfunction in the pathophysiological process of SEs.

In the study described above, the patient with MELAS underwent double brain PET imaging with ^62^Cu-ATSM and ^18^F-fluorodeoxyglucose (^18^F-FDG) to evaluate oxidative stress and the glucose metabolism, respectively [10]. At the time of the PET scans, the patient had three SE lesions: An acute lesion that occurred one day before the scans, a subacute lesion that developed one month before, and a chronic lesion that appeared half a year before. ^18^F-FDG PET demonstrated increased uptake in the acute lesion, but the subacute and chronic lesions showed decreased uptake. In contrast, ^62^Cu-ATSM PET revealed significant accumulation in the subacute lesion (1.8-fold SUV increase compared with that in the acute lesion) (Figure 2). The acute lesion showed slightly increased uptake of ^62^Cu-ATSM, and decreased uptake was observed in the chronic lesion. These findings indicate that enhanced glycolysis is followed by increased oxidative stress due to an over-reductive state, which eventually leads to neuronal cell death in SEs [10]. Combined with the results of imaging studies, we proposed the pathophysiological process of SEs as Figure 2 [10,74]. In addition to elucidating the pathogenesis of SEs, this PET study demonstrates the feasibility of ^62^Cu-ATSM PET for the evaluation of cerebral oxidative stress based on mitochondrial dysfunction in living patients.

## 5. Oxidative Stress Imaging in Parkinson’s Disease

### 5.1. Oxidative Stress and Mitochondrial Dysfunction in Parkinson’s Disease

**Figure 3:**
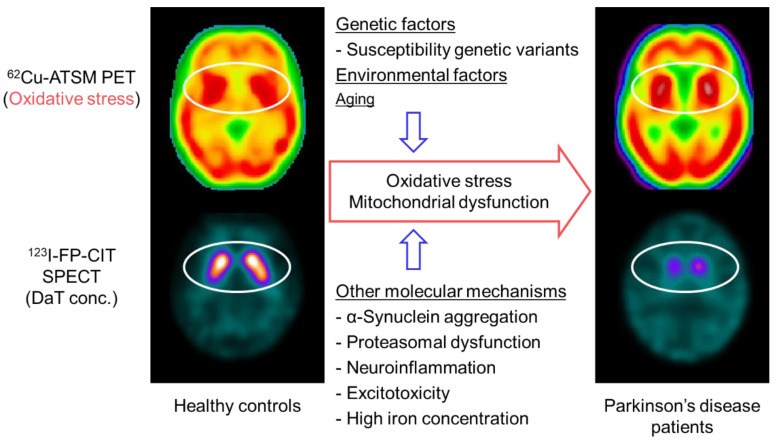
Brain positron emission tomography (PET) imaging with ^62^Cu-ATSM for oxidative stress and putative molecular mechanisms in patients with Parkinson’s disease. Single-photon emission computed tomography (SPECT) with ^123^I-FP-CIT, which reflects the density of dopamine transporters (DaT) in the striatal dopaminergic nerve terminals, showed decreased uptake in the striatum of patients with Parkinson’s disease compared with that of healthy controls (circles in the lower images). In contrast, ^62^Cu-ATSM PET revealed increased striatal accumulation in patients with Parkinson’s disease compared with healthy controls, which suggests enhanced oxidative stress in the striatal dopaminergic neurons in Parkinson’s disease (circles in the upper images).

Parkinson’s disease is the most common motor neurodegenerative disorder, with a prevalence of more than 1% in elderly individuals [75]. The pathological findings are characterized by the degeneration of dopaminergic neurons in the nigrostriatal system with the appearance of Lewy bodies composed of α-synuclein aggregation in the remaining neurons [76]. While various pathogenetic molecular mechanisms as well as genetic and environmental factors are believed to be associated with the cause and pathogenesis, a number of pathobiochemical and genetic studies have indicated that oxidative stress and mitochondrial dysfunction play a major role in the nigrostriatal neurodegeneration in Parkinson’s disease (Figure 3) [77,78].

Since the endogenous dopamine metabolism with a high iron concentration in the nigrostriatal neurons inevitably induces auto-oxidation of dopamine to generate dopamine quinones and ROS [79,80], the involvement of oxidative stress in the pathogenesis of Parkinson’s disease has been assumed. In fact, multiple postmortem studies showed increases in the levels of 8-OHdG, protein carbonyls, 4-hydroxy-2-nonenal (4-HNE) histidine, or lipid peroxidation products (i.e., oxides of DNA, proteins, and lipids, respectively) in the nigrostriatal system of patients with Parkinson’s disease [81,82,83]. Changes in the levels of antioxidant molecules, such as manganese SOD and GSH, were demonstrated in other pathological studies [84,85]. Interestingly, a recent study using a mouse model with α-synuclein overexpression showed that oxidative stress induced by paraquat exposure resulted in the aggregation and propagation of α-synuclein, and marked neurodegeneration [86]. These findings suggest that oxidative stress contributes to the pathological processes of Parkinson’s disease.

The involvement of mitochondrial respiratory dysfunction in the pathogenesis of Parkinson’s disease has been strongly suggested by postmortem and in vivo model studies [77,87], such as activity deficiency of the mitochondrial respiratory chain complex I in the substantia nigra of patients and an animal model induced by 1-methyl-4-phenyl-1,2,3,6-tetrahydropyridine (MPTP), an inhibitor of complex I [88,89,90]. In addition, genetic studies have revealed that many causal genes of monogenic Parkinson’s disease, such as *Parkin* (PARK2) [91], *PINKl* (PARK6) [92], *DJ-1* (PARK7) [93], *Omi/HtrA2* (PARK13) [94], and *CHCHD2* (PARK22) [95,96], are associated with mitochondrial functions and quality control [77,97]. Especially, PINK1 recruits Parkin to induce autophagic degradation of the impaired mitochondria with low membrane potential (i.e., mitophagy), which suggests that these genetic products are instrumental for mitochondrial quality control [98,99].

Similar to Parkinson’s disease, a recent study revealed *COQ2* gene mutations in patients with familial multiple-system atrophy, which presents with Parkinsonian symptoms due to nigrostriatal degeneration [100]. COQ2 is essential for the biosynthesis of coenzyme Q_10_ [101], a key component in mitochondrial respiratory chains, which also indicates the involvement of mitochondrial dysfunction in the dopaminergic neurodegeneration. Thus, these pathological and genetic studies strongly suggest that mitochondrial respiratory chain impairment leading to oxidative stress is one of the principal pathogenetic mechanisms in Parkinson’s and its related diseases. However, the effects of oxidative stress due to mitochondrial failure on the neurodegeneration process in living patients remain unknown.

### 5.2. PET Imaging for Oxidative Stress in Patients with Parkinson’s Disease

Based on the oxidative stress hypothesis, a PET study with ^62^Cu-ATSM was performed to investigate cerebral oxidative stress in patients with sporadic Parkinson’s disease [11]. This PET study found a significant increase in ^62^Cu-ATSM uptake in the bilateral striata in the patient group as compared to that in healthy subjects (6% increase in the striatum-to-cerebellum SUV ratio) (Figure 3). The uptake in the striatum was positively correlated with the clinical severity in patients, as estimated by the Unified Parkinson’s Disease Rating Scale (UPDRS) score. These findings strongly indicate the involvement of oxidative stress based on mitochondrial dysfunction in the neurodegeneration process in Parkinson’s disease [11,13]. However, the density of the remaining nigrostriatal dopaminergic neurons decreases markedly with the progression of Parkinson’s disease [102], which may offset the striatal uptake of ^62^Cu-ATSM in patients with advanced Parkinson’s disease.

To resolve this problem, single-photon emission computed tomography (SPECT) with ^123^I-N-ω-fluoropropyl-2β-carbomethoxy-3β-(4-iodophenyl)nortropane (^123^I-FP-CIT) was performed along with ^62^Cu-ATSM PET in patients with Parkinson’s disease in another study (Figure 3) [103]. ^123^I-FP-CIT accumulates in the dopamine transporters that are highly expressed in the striatal dopaminergic nerve terminals. The striatal ^123^I-FP-CIT binding thereby approximately reflects the density of the remaining striatal pre-synaptic dopaminergic neurons [104]. Thus, the ^62^Cu-ATSM uptake levels corrected by ^123^I-FP-CIT binding levels for the striatum may precisely indicate oxidative stress in the remaining striatal neurons. The corrected ^62^Cu-ATSM uptake significantly increased with disease severity, as estimated by the UPDRS scores in patients with Parkinson’s disease, when compared with the non-corrected ^62^Cu-ATSM uptake [103]. These results suggest that oxidative stress is enhanced in the remaining dopaminergic neurons, which may facilitate the dopaminergic neurodegenerative process even in advanced Parkinson’s disease.

According to the above substantial evidence implicating oxidative stress based on mitochondrial dysfunction in the pathogenesis of Parkinson’s disease, many pharmaceutical molecules having effects of oxidative stress reduction and/or mitochondrial function improvement have been developed to modify the disease course [78]. While only a few agents, such as inosine (a precursor of urate) and the reduced form of CoQ_10_, have shown possible therapeutic efficiency in patients with Parkinson’s disease [105,106,107], the results of ^62^Cu-ATSM PET studies suggest that optimized antioxidative agents would be effective even in the advanced disease stage. Interestingly, Hung et al. showed the therapeutic effects of non-radioactive Cu-ATSM in multiple animal models of Parkinson’s disease [108]. Rodent models treated with oral administration of Cu-ATSM exhibited restoration of motor function and dopamine biosynthesis and the prevention of nigral dopaminergic cell death. Besides its utility as an imaging ligand for oxidative stress, these findings suggest the therapeutic potential of Cu-ATSM as an antioxidant in Parkinson’s disease.

### 5.3. PET Imaging for Neuroinflammation and Mitochondrial Activity in Parkinson’s Disease

Neuroinflammation mediated by the activated microglia is another principal molecular mechanism of the neurodegenerative process [109]. Oxidative stress and mitochondrial dysfunction are induced by neuroinflammation [110,111]; conversely, ROS induces inflammation via the activation of NF-κB [15], which suggests an inextricable relationship between these pathological factors [7]. Several PET studies with radioligands for 18-kDa translocator protein (TSPO), which is highly expressed in activated microglia in the brain [112], such as ^11^C-(*R*)-PK11195 and ^11^C-DPA713, revealed increased cortical uptake in patients with Parkinson’s disease, especially in the early stage of the disease [113,114,115]. While recent PET studies with other TSPO radioligands, ^11^C-PBR28 and ^18^F-FEPPA, showed conflicting results [116,117], the PET studies that yielded positive results indicated that neuroinflammation due to microglia activation occurs in the early phase of neurodegeneration in Parkinson’s disease [113,114,115]. These findings of neuroinflammation in the early stage contrast with the ^62^Cu-ATSM PET studies showing that oxidative stress increases with disease progression [11,13,103]. The relationship between oxidative stress and neuroinflammation in the pathophysiological process of Parkinson’s disease requires further clarification [118]. Interestingly, Metzger et al. showed increased uptake of both ^11^C-PBR28 and ^61^Cu-ATSM in the myocardium one week after systemic administration of 6-hydroxydopamine (6-OHDA), which causes cardiac catecholaminergic neuronal degeneration due to induction of oxidative stress and inflammation, in non-human primates [119]. While it was a cardiac investigation, this study suggested that oxidative stress and neuroinflammation arise concurrently, at least in the acute intoxication model.

Recently, a promising PET radioligand, ^18^F-BCPP-EF, for imaging of the activity of mitochondrial complex I has been developed [120]. A preclinical PET study with ^18^F-BCPP-EF showed decreased uptake in the cortex and basal ganglia in non-human primates treated with MPTP as an animal model of Parkinson’s disease [121]. While clinical studies with ^18^F-BCPP-EF PET are still ongoing [122], a direct comparison between mitochondrial activity and oxidative stress is anticipated in patients with Parkinson’s disease using these PET imaging techniques.

## 6. Oxidative Stress Imaging in Amyotrophic Lateral Sclerosis (ALS)

### 6.1. Oxidative Stress and Mitochondrial Dysfunction in ALS

**Figure 4:**
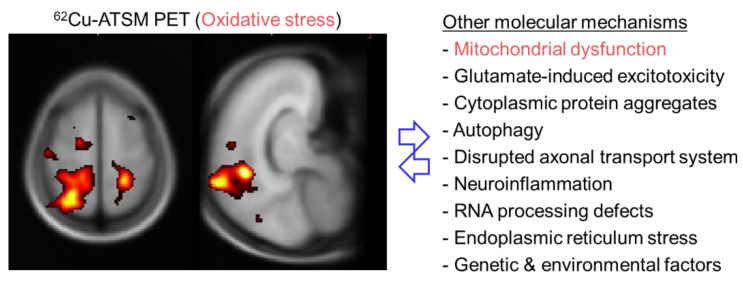
Brain positron emission tomography (PET) imaging with ^62^Cu-ATSM for oxidative stress and putative molecular mechanisms in patients with amyotrophic lateral sclerosis (ALS). The left panel shows the T-map generated by statistical parametric mapping analysis, displaying the regions in which ^62^Cu-ATSM accumulation was higher in patients with ALS than in healthy controls. A significantly greater accumulation of ^62^Cu-ATSM in patients with ALS than in controls was observed in the bilateral cortices around the central sulcus, including the motor cortex, and the right superior parietal lobule. These results demonstrate increased oxidative stress, primarily in the motor cortex, in patients with ALS.

ALS is an intractable neurodegenerative disorder characterized by progressive degeneration of both upper and lower motor neurons [123]. Various pathophysiological mechanisms have been assumed to be involved in the pathogenesis of ALS, including glutamate-induced excitotoxicity, cytoplasmic protein aggregates, autophagy, disrupted axonal transport system, inflammation caused by activated microglia, RNA processing defects, and endoplasmic reticulum stress (Figure 4) [123,124]. In addition to these factors, a number of investigations have indicated that oxidative stress based on mitochondrial dysfunction plays a principal role in the motor neuron degeneration in ALS [125,126]. Multiple postmortem studies have demonstrated the accumulation of cellular component oxides (i.e., DNA, proteins, and lipids) indicated by increases in the levels of 8-OHdG, protein carbonyls, or 4-HNE histidine in the motor cortex and/or spinal cord in patients with ALS [127,128,129,130]. Similarly, biochemical studies showed increased concentrations of 8-OHdG or 4-HNE in patients’ blood, urine, or cerebrospinal fluid [131,132,133]. Genetic factors, especially *SOD1* and *TDP-43* gene mutations frequently found in familial ALS, also indicate the involvement of oxidative stress in the pathogenesis [134]. Linked with oxidative stress, postmortem investigations showed mitochondrial alterations and mitochondrial respiratory chain impairment in the spinal cord or muscles in patients with ALS [135,136]. Mitochondrial abnormalities were also observed in transgenic mice and cell culture models carrying *SOD1* gene mutations [137]. These biopathological and genetic studies provide collateral evidence, but cannot directly evaluate regional changes of the redox status in living patients with ALS.

### 6.2. PET Imaging for Oxidative Stress in Patients with ALS

To evaluate cerebral oxidative stress and its relationship with the clinical features, a PET study with ^62^Cu-ATSM was performed in patients with sporadic ALS [12]. This study demonstrated a significantly greater accumulation of ^62^Cu-ATSM in patients with ALS than in healthy controls, mainly in the bilateral cortices around the central sulcus, including the motor cortex and motor-related parietal areas (9% SUV increase after global normalization in the bilateral cortices around the central sulcus) (Figure 4). Furthermore, ^62^Cu-ATSM accumulation in these regions correlated positively with the clinical severity in patients, as estimated by the revised ALS Functional Rating Scale (ALSFRS-R) score [12]. These findings indicated that increased oxidative stress in motor and motor-related cortices strongly correlated with the disease severity in patients with ALS, which is consistent with biochemical studies showing that serum 4-HNE or urine 8-OHdG levels increased with clinical severity in patients with ALS [131,133]. Thus, the results of ^62^Cu-ATSM PET imaging successfully indicated that oxidative stress based on mitochondrial dysfunction is associated with motor neuron degeneration in ALS.

Focusing on the involvement of oxidative stress in the pathogenesis of ALS, some therapeutic agents showing antioxidant effects have been developed [138]. The therapeutic efficacy of edaravone, a free radical scavenger, has been demonstrated in patients with ALS [139,140]. In addition, similar to Parkinson’s disease, several therapeutic studies yielded positive outcomes of non-radioactive Cu-ATSM in *SOD1*-mutant mouse models of ALS, including improved locomotive function and overall survival [141,142]. Cu-ATSM has the potential to detoxify mutant SOD1 by supplying Cu, as well as antioxidant activity inhibiting lipid peroxidation and iron accumulation (i.e., ferroptosis), which may result in neuroprotective effects [143,144,145]. Based on these basic studies, a Phase 2 clinical trial using Cu-ATSM in patients with ALS commenced in Australia in 2019 (NCT04082832). Thus, in addition to its imaging utility, Cu-ATSM is also a potential therapeutic agent for ALS [146].

### 6.3. PET Imaging for Other Factors Associated with Oxidative Stress in ALS

Besides oxidative stress, many pathological molecular factors have been investigated using PET imaging in patients with ALS [147,148]. Interestingly, several PET studies to image neuroinflammation using TSPO radioligands, such as ^11^C-(*R*)-PK11195, ^18^F-DPA714, and ^11^C-PBR28, showed increased uptake mainly in the primary motor cortex in ALS patients [149,150,151,152]. Taken together with the ^62^Cu-ATSM PET results, this indicates that oxidative stress and neuroinflammation may occur concurrently in the brain and may contribute together to neurodegeneration in ALS.

In contrast to neuroinflammation, ^18^F-FDG PET studies showed a decreased glucose metabolism in the primary motor cortex in patients with ALS [153,154,155]. Based on the aforementioned PET study in MELAS showing increased ^18^F-FDG uptake followed by elevated ^62^Cu-ATSM accumulation in SE lesions [10], a period of enhanced oxidative stress may be inconsistent with that of hypermetabolism in the cerebral cortex. Unlike the upper motor neurons of the motor cortex, several studies showed increased ^18^F-FDG uptake in the brainstem and spinal cord, which include lower motor neurons, in patients with ALS [153,155,156,157]. The uptake value correlated positively with disease progression, which suggests a difference in metabolic mechanism between these regions and the cerebral cortex [155].

## 7. Application of ^62^Cu-ATSM PET to Cerebrovascular Diseases

### 7.1. Pathophysiological Changes in Brain Misery Perfusion

**Figure 5:**
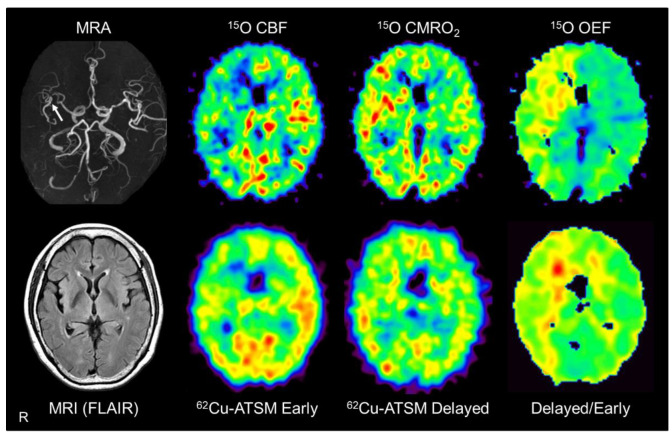
Brain positron emission tomography (PET) imaging with ^62^Cu-ATSM and ^15^O tracers (^15^O-water, ^15^O_2_, and C^15^O) in a representative patient with major cerebral arterial occlusive disease. Brain magnetic resonance imaging (MRI) showed severe stenosis in the right middle cerebral artery (arrow) but no apparent infarction. PET images with ^15^O tracers (upper images) showed decreased cerebral blood flow (CBF) with increased cerebral metabolic rate of oxygen (CMRO_2_) and oxygen extraction fraction (OEF) in the right cerebral hemisphere, which indicates misery perfusion in this area. Alongside ^15^O-gas and water PET, early, delayed, and delayed-to-early ratio images from ^62^Cu-ATSM PET (lower images) correspond well with the CBF, CMRO_2_, and OEF images, respectively. MRA: MR angiography; FLAIR: Fluid attenuation inversion recovery.

Chronic steno-occlusive changes in the major cerebral arteries are known to be a risk factor for ischemic cerebral infarction [158]. Especially, the region with “misery perfusion” that exhibits decreased cerebral blood flow (CBF) relative to normal oxygen consumption shows a high risk of infarction [159]. In misery perfusion, the OEF is increased to sustain the aerobic energy metabolism in mitochondria (i.e., CMRO_2_) (Figure 5); however, chronic ischemic changes gradually induce mitochondrial dysfunction [160,161]. Thus, enhanced OEF with mild mitochondrial respiratory chain impairment may induce an over-reductive state in chronic misery perfusion, which would ultimately lead to oxidative stress and neuronal damage [158,161]. To precisely detect misery perfusion carrying a high risk of exacerbation, the assessment of hemodynamic parameters including OEF and CBF is indispensable [159].

### 7.2. PET Imaging in Patients with Misery Perfusion

Based on the above assumption, PET scans with ^62^Cu-ATSM and ^15^O tracers (^15^O-water, ^15^O_2_, and C^15^O) performed in patients with unilateral steno-occlusive lesions in the major cerebral arteries were compared [52]. This double PET study showed that the delayed-to-early ratio of ^62^Cu-ATSM correlated significantly with the OEF value derived from PET with ^15^O tracers in these patients (Figure 5). In addition, the early-phase accumulation of ^62^Cu-ATSM correlated well with the CBF value obtained from ^15^O-gas and water PET, as expected (see Section 3). While ^15^O-gas and water studies can provide accurate CBF and OEF values [160,161], PET imaging with ^15^O tracers has some disadvantages, such as the need for invasive arterial blood sampling and cumbersome handling of the tracers. The results of the ^62^Cu-ATSM PET study indicate the feasibility of a simple PET scan with a single injection of ^62^Cu-ATSM for detecting chronic misery perfusion with increased OEF and reduced blood flow [52].

## 8. Recent Progress and Perspective of Oxidative Stress Imaging

### 8.1. PET Imaging for Oxidative Stress in Patients with Alzheimer’s Disease

Alzheimer’s disease is the most common neurodegenerative disorder causing progressive dementia. The pathological findings of extracellular deposits of amyloid-β plaques and intraneuronal neurofibrillary tangles containing aggregated tau protein are observed as diagnostic markers in the brains of patients with Alzheimer’s disease [162,163]. PET imaging with radioligands for amyloid-β or tau protein, such as ^11^C-PiB and ^18^F-MK-6240, respectively, is already being used to diagnose Alzheimer’s disease in living patients [164,165,166], replacing pathological diagnosis.

While various pathophysiological mechanisms including genetic and environmental factors have been proposed for the pathogenesis of Alzheimer’s disease [163], a number of investigations using postmortem specimens or serum samples have emphasized the involvement of oxidative stress and mitochondrial dysfunction, similar to other neurodegenerative disorders [6,167,168]. In support of this hypothesis, a recent PET study with ^18^F-BCPP-EF, a radioligand to image mitochondrial complex I activity, demonstrated decreased uptake in the parahippocampus in patients with early-stage Alzheimer’s disease [169], which supports the involvement of mitochondrial dysfunction.

We are engaged in PET imaging for oxidative stress using ^64^Cu-ATSM, instead of ^62^Cu-ATSM, combined with PET imaging with ^11^C-PiB and ^18^F-MK-6240 for amyloid-β and tau protein, respectively, in patients with Alzheimer’s disease. ^64^Cu-ATSM has a longer radioactive half-life (~13 h) than ^62^Cu-ATSM (~10 min), and is thus expected to more precisely evaluate cerebral oxidative stress [170]. By combining these PET imaging protocols, investigation of the relationships among protein accumulation, disease progression, and oxidative stress would shed light on the molecular mechanisms of Alzheimer’s disease.

### 8.2. Development of Imaging Techniques for Oxidative Stress

Besides ^62^Cu-/^64^Cu-ATSM, several PET radioligands for imaging of the redox status have been developed, such as ^18^F-FASu for the cystine/glutamate transporter [171], ^18^F-ROStrace for superoxide [172], and ^18^F-FDHM for ROS [173]. These new radioligands are promising but are still in the preclinical stage with animals. In addition to PET, a new MR imaging method using hyperpolarized ^13^C-MR spectroscopy permits measurement of the reduction rate of 1-^13^C-dehydroascorbic acid in tumor cells, which reflects the capacity of tumor cells to resist oxidative stress [174,175]. Several MR contrast agents, such as paramagnetic nitroxide radicals (e.g., mito-TEMPO, 3-carbamoyl-PROXYL) and paramagnetic chemical exchange saturation transfer agents, have also been developed to evaluate the redox status in vivo [176,177,178]. As new MR techniques for imaging oxygen metabolism advance, the OEF and oxygen metabolism, as well as oxidative stress, will also be elucidated by MR imaging [179]. However, PET has the advantage of higher detection sensitivity at the nanomolar level [14], which suggests that the optimal approach will be multimodal imaging combining PET and MR [165,180]. Imaging techniques for oxidative stress will further develop in support of clinical research aimed at clarifying the pathophysiological mechanisms of various neurodegenerative disorders.

The use of PET imaging is not limited to elucidating the pathogenesis; it can also directly evaluate the effects of therapeutic agents on the pathological targets. In addition to the aforementioned studies in Parkinson’s disease and ALS, several clinical trials testing new therapeutic molecules having antioxidant effects, such as vatiquinone (EPI-743) and elamipretide (MTP-131), are ongoing on patients with mitochondrial diseases [181,182]. Oxidative stress imaging is thus also able to assess the therapeutic efficacy of these agents against oxidative stress in living patients.

## 9. Conclusions

This review provides an overview of the availability and future potential of recent imaging techniques for oxidative stress, especially ^62^Cu-/^64^Cu-ATSM PET, in neurodegenerative disorders. Clinical ^62^Cu-ATSM PET studies showed increased uptake in brain regions of pathologically responsible sites of neurodegeneration, i.e., the SE lesions of mitochondrial disease (MELAS), the striatum of Parkinson’s disease, and the motor and motor-related cortices of ALS [10,11,12,13]. These PET studies delineated enhancement of oxidative stress in the disease-related brain regions, suggesting that oxidative stress based on mitochondrial dysfunction is closely associated with the neurodegenerative process in these diseases. Similarly, future studies with oxidative stress and neuroinflammation imaging would also focus on the cardinal brain pathophysiology of dementia and other neurodegenerative disorders, such as amyloid and tau deposition in Alzheimer’s disease. PET imaging for oxidative stress improves our insight into the pathogenesis of neurodegenerative disorders, and is a promising tool for monitoring further antioxidant and mitochondrial therapies.
